# Extracts and Gleanings

**Published:** 1872-02

**Authors:** 


### PART II.

### Abridged Extracts and Gleanings from our Exchanges

*MULTUM IN PARVO.*

A SOVEREIGN ANTI-BILIOUS PILL.—We transcribe (by permission) from the Prescription-Book of one who has been actively engaged in practice for the last 30 years, a formula for an Anti-Bilious Pill, which, we feel confident, will find favor with all disposed to adopt it. As will be seen, besides containing all the components of the "Cook Pill"—a pill of established popularity in the South—it also embodies other important adjuvants. The gambage and podophyllin gives increased action and augmented hepatic tendency, whilst the capsicum and extract hyosciamus impart to the whole a fine corrective and anti-drastic quality. By substituting pure castile soap for the calomel, we also have a pill that will effect all that is meant by a "Vegetable Anti-Bilious Pill:"

| | | |
|---|---|---|
| R.—Calomel, Rheubarb, Aloes, | aa. . . | dr. j. |
| Capsicum, Gamboge, Podophyllin, Ext. Hyosciamus, | aa. . . | gr. xij.—M. |

Make mass with Ar. Syr. Rheubarb, and divide into 22 pills. Dose from 2 to 4 for audlts, as a prompt and efficient anti-bilious evacuant.

J. KNOX HODGE, M. D.
Holly Springs, Ark.

CANCRUM ORIS.—Prof. Byford, (Chicago Medical Examiner) says: "The treatment is to touch all the advanced ulcers with nitric acid in order to get a new and plastic inflammation. Tonics and nutritious food are to be given in as large quantites as the patient can take. Quinine, gr. ij., is to be given every four or six hours, and a dressing of carbolic acid and glycerine applied.

Erysipelas.—Prof. N. S. Davis, (Chicago Medical Examiner,) in an able an interesting article on erysipelas, gives the following as his method of treating the disease:

"In a much larger number of cases, in which the abstraction of blood is not necessary, there is much benefit derived from giving at the commencement five grains of calomel, with five of bi-carbonate of soda, and if they do not cause a movement of the bowels in four or five hours, aiding them by a saline laxative. Immediately after the proper evacuations, I commence the use of some one of the remedies that are regarded as more directly curative in their influence over the disease. Abundant opportunities for observation have satisfied me that the tincture of chloride of iron, and the sulphites of soda and lime, are capable of exerting as specific an influence over the progress of idiopathic erysipelas, as quinine is over the paroxysms of an intermittent fever. And in a large majority of the cases, as they are presented to the practitioner, no preparatory treatment by evacuations or alteratives, is necessary, before commencing the use of this class of remedies. If the tincture of chloride of iron is relied on, it should be given to adults in doses of from 20 to 30 drops, in half a wine-glassful of sweetened water, every two or three hours, according to the severity of the case. After the first forty-eight hours, the interval between the doses may be increased to three or four hours, and continued at that rate until the redness and swelling have disappeared, and convalescence is fully established. Under this treatment the disease generally ceases to extend its boundaries after the third day, and by the seventh, convalescence is fairly begun. In some cases a mild laxative may be needed two or three times during the progress of the case; and in other instances, the bowels will become relaxed in the advanced stage, needing the exhibition of five or six grains of Dover's powder each night.

In a few cases the tincture of chloride of iron has disturbed the stomach sufficiently to cause its rejection by vomiting. When this happens, the most reliable substitute is the sulphite of soda, in doses of from 10 to 20 grains, dissolved in a small wine-glassful of water, and repeated every two, three or four hours. In the epidemic of 1863, I tested the efficiency of the sulphites of soda and lime, in several very severe cases, and was well satified with the results. During the same season six of the most severe cases admitted into the Mercy Hospital, were complicated with a typhoid grade of dysentery. To these I gave twenty grain-doses of sulphite of lime mixed in a table-spoonful of sweet milk, every four hours, and ten drops each of oil of turpentine and tincture of opium in a tea-spoonful of an emulsion of gum-arabic and sugar, half-way between the doses of the sulphite. The uniform result was a speedy improvement in the symptoms and an early convalescence.

When erysipelas occurs in a malarious district, the fever accompanying it exhibits, generally, more or less of a periodical character. In such cases the same remedies should be given, with the addition of anti-periodic doses of sulphate of quinine at each remission, in the early stage; and smaller doses may be combined with the iron in the later stage.

CHRONIC OSTITIS.—Dr. W. L. Lipscomb, Columbus, Miss., (Med. and Sur. Reporter,) says, his favorite prescription is:

R.—Argent nit. fused, . . . gr. xl.
Aquæ, . . . . . . f. oz. j.—M.

Sig.—Apply twice a week, with c. h. pencil.

He reported several cases thus treated with perfect success.

AMENORRHŒA SUCCESSFULLY TREATED BY ELECTRICITY.—Dr. Minot reports a case (Boston Medical and Surgical Journal):

The patient was a young lady, in whom amenorrhœa had existed for seven months. A current was passed from hand to hand, and from one hand to the opposite foot. This was repeated daily for three consecutive days; the catamenia appeared on the fourth day.

The form of electricity used was obtained from Farmer's thermo-electric battery. In obstinate cases, Dr. Minot applied one pole over the sacrum and the other over the pubes. It should be employed at or near the menstrual period.

AURAL POLYP.—A well developed boy, æt. 12 years, is brought to the clinic by his mother, on account of a discharge from the ear, which followed an attack of scarlet fever, incurred at the age of four years. When the discharge, which is scant and offensive, commenced, the attending physician told the mother to let the boy alone as he would outgrow it.

Prof. Gross said that he was not a believer in the outgrowing of diseases. Every practitioner is called upon, when a disease is presented to him, to study alike its etiology and pathology, and endeavor to sap it as it exists. "I have poor children brought to me constantly, with paralysis, and spine disease, and white-swelling, and as constantly the statement of some former attention, 'let the child alone, it will outgrow it.' In this age of enlightenment this should not be!"

An examination of the boy now revealed the drum of the ear, on the affected side, completely destroyed; and growing from one side of the auditory meatus by a narrow pedicle was a kind of papillary tumor, or mass of granulations composed of connective tissue. This growth, or polyp, was probably primarily dependent on some disease of the bone. It was twisted off with the forceps, and to prevent its reproduction, its base was touched with the solid nitrate of silver. The ear was directed to be syringed with luke-warm water, and also,

R.—Potass. Permangan., - - grs. xxx.
Aquæ, - - - - - oz. iv.—M.

S.—Add a tea-spoonful to half a tumblerful of water, and inject in the ear two or three times daily.

*November* 22.—This patient again reports at the clinic. There was either a little bit of the tumor left at the first operation or some has been reproduced. It was removed with the forceps as before. The discharge since the operation has not been so great, neither has the boy been alike repulsive to himself and those around him by reason of its bad odor. The injection was ordered to be continued, and the parts touched every other day with a ten-grain solution of the nitrate of silver.—*Med. and Sur. Reporter.*

Cerebro-Spinal Meningitis.—Dr. Williams, (proceedings of the Baltimore Medical Association—Med. and Sur. Reporter,) in speaking of the use of opium in this disease, says—quoting from Dr. Stille:

"Strong is the first who alludes to its use to relieve coma, and he urges that if the medicine is rejected by the stomach it should be administered by the rectum. 'Opium,' he remarks, 'either pure, or in the form of laudanum, was found a most excellent stimulant in every stage of this disease.'

The best manner of exhibiting it appeared to be in small doses often repeated, so that the system should be constantly under a gentle influence from it. It tended to relieve pain, increase the excitement, and remove delirium and stupor. The necessary dose varied according to the mildness or violence of the symptoms, from ten drops of laudanum every hour to thirty drops every two hours. In those cases of the disease especially, which commenced with sudden and violent delirium, this medicine exceeded all others in its beneficial effects. In such cases, however, the dose required was often large, but when exhibited with great freedom, it produced the happiest and most striking effects.

A case is then related, in which excruciating agony in the head and maniacal delirium were predominant symptoms. Sixty drops of laudanum were administered every hour until 480 drops, or half a fluid ounce had been taken in the course of eight hours. The whole of it was retained, and it subdued the excitement and allayed the pain, but produced no sleepiness, nor any other apparent effect of opium. In a like heroic manner, Haskell employed this remedy. He speaks of a young woman who recoved, but who, during her illness, took more than a quart of brandy and not less than twenty grains of good Turkey opium, and that without any evidence of intoxication. 'Indeed,' he adds, 'we have been obliged frequently to exhibit ten grains of opium for a dose in some of the violent cases attended with strong spasms, and have never known it to produce stupor in a single instance." So Minor states that 'a few cases imperiously required half an ounce of the tincture in an hour, or half a drachm in substance in the course of twelve hours before the urgent symptoms could be controlled, and even some cases required a drachm in the same time. All these patients recovered."

Amaurosis.—Dr. Waring relates the following case in his therapeutics, p 117, taken from the Med. Chir. Rev., July 1, 1842:

"In Amaurosis, Arnica has long been a popular remedy in Germany. M. Maumoir, of Geneva, relates an obstinate case which completely yielded to the following formula:

| | | |
|---|---|---|
| R.—Ext. Arnica, | . . . . | oz. ij. |
| Strychniæ Sulph., | . . . . | gr. xii. |
| Conf. Rosæ, | . . . | q. s. ft. pil. cxi. |

Dose, one every night, gradually increased, until five are taken daily. The latter dose created much irritation."—*Jour. Materia Medica.*

Broncho-Pneumonia, with Typhoid Symptoms.—Prof. Davis (Chicago Med. Examiner,) gave the following treatment:

"To a patient in the second week of typhoid fever, with a pulmonary complication which made his condition quite serious. The expectoration had been tinged with blood for two or three days, and there was dullness over a part of one lung with severe bronchial cough. These symptoms led to the conclusion that the inflammatory action, which had existed in the capillary bronchial tubes was extending to some of the lobules of the lungs. The typhoid depression was considerable and the indications were to sustain the circulation and innervation, and to induce freer secretion from the pulmonary mucous membranes, and thus lessen the tumefaction and admit a larger supply of oxygen to the blood. The first indication is well met by the following prescription:

℞.—Ammonia Carbonate, . . . gr. iv.
Quinia Sulph., . . . . . gr. ij.
Camphor Gum, . . . . gr. j.

Take every four hours.

To accomplish the second object, the mixture of muriate of ammonia, tartrate of antimony and morphia, in syrup of liquorice, was given every four hours, alternating with the other medicine. A blister was also applied to the sternum. At present the pulse is fuller and less frequent, the expectoration more abundant, and the whole aspect of the case decidedly better. Four days later the pulmonary symptoms had nearly disappeared, and the fever was better.

TYPHOID FEVER WITH CAPILLIARY BRONCHITIS.—Prof. N. S. Davis (Chicago Med. Examiner) treated a case as follows:

"This is a case of typhoid fever complicated with capillary bronchitis, which has been on the use of the emulsion of turpentine and laudanum and the muriate of ammonia mixture. The former medicine has brought the bowels into a satisfactory condition, but the want of innervation has been so great that the patient was in much danger from the depression of the nerve centers. To remedy this he was given the following prescription:

℞.—Strychnia, . . . . gr. j.
Acid, nitric. . . . . dr. j.
Tinct. opii, . . . . dr. iij.
Aquæ, . . . . . oz. jv.—M.

Dose—One tea-spoonful in sweetened water every four hours.

Now, after two days, the patient is improved, the countenance looking better and the mind clearer; but the typhoid depression is still considerable, and it is best that the tonic be continued. The muriate of ammonia mixture is also to be continued on account of the bronchitis. On examining the chest the spaces above the clavicles and between the ribs are seen to sink at the beginning of each inspiratory act and to bulge during expiration. This is evidently due to the difficulty in getting air into the air cells on account of the obstruction of the capillary tubes; on the expansion of the chest the air cannot enter fast enough to fill the cells, and a partial vacuum is produced; atmospheric pressure from without then presses in the soft tissues at the intercostal spaces; while in expiration the air is, with equal difficulty, passed out of the cells through the obstructed tubes. Thus forcible inspiration and slow expiration is the charateristic respiration where the air cells are themselves permeable but the capillary tubes partially closed. But when the air cells are infiltrated the respiratory acts are short.

Sometimes when there is typhoid depression, wandering of the mind and bronchial tightness, chloroform is useful, and in this case may be given with propriety according to the following formula:

| | |
|---|---|
| R.—Chloroform, . . . . | dr. iij. |
| Accaciæ, . . . . . | dr. vj. |
| Sacchari, . . . . . | dr. vj. |
| Aquæ, . . . . . . | oz. vj.—M. |

Dose—A table-spoonful every four hours, alternately with the other medicine.

Remedy for Dandruff.—J. L. Davis (Am. Jour. of Pharmacy) speaks highly of the following: He takes one ounce of flowers of sulphur to one quart of water—the clear liquor is poured off after the mixture has been agitated repeatedly during intervals of a few hours. The head is to be saturated with this every morning. In his own case, in a few weeks, every trace of dandruff had disappeared, the hair becoming soft and glossy.

Chronic Cystitis.—Dr. Theophillus Parvin, in the Clinic, gives an account of a vesico-vaginal fistula made for the cure of chronic cystitis. He says: "Thus far the condition of the patient has been decidedly improved, and there is every reason to hope that, in the course of six or eight months, by the *rest* given to the bladder and the faithful use of warm water injections, the cystitis will be cured, and then the fistula can be readily closed."

Cure for Corns.—Bathe the feet well in warm water, then with a sharp instrument pare off as much of the corn as can be done without pain or causing it to bleed, and dress once a day with the following salve:

| | |
|---|---|
| R.—Black Oxide of Copper, . . | gr. xv. |
| Lard, . . . . . . | oz. ss.—M. |

—*Chem. and Drug., Lond., Nov.* 15, 1871.

Chloroform and Glycerine.—Dr. W. Murdock, of New York, recommends the following formula as a convenient mode of administering chloroform: Glycerine, six ounces; chloroform, two ounces. This solution is clear, and not unpleasant in taste or ordor. One drachm contains fifteen minums of chloroform.

Formulæ in Local Use.—Mr. Louis S. Cohen, N. Y., (Am. Jour. of Pharmacy,) publishes the following formulæ in local use in New York City, and which he highly values:

"Believing it to be the duty of every conscientious pharmacist to contribute, through the Pharmaceutical Journals, to the profession whatever improvements he may be able to make, be they ever so trifling, I cannot approve of the practice of some in our ranks who make certain preparations, bestowing on them, with quite an air of authority, *officinal titles*, and regard them as proprietary specialties in claiming superiority for their formulæ, without giving the profession the benefit thereof. Some pharmacists have endeavored to publish all their observations that may be useful to others; yet they are "few and far between." By following such examples, our science and art would more readily be raised to a far higher standard than it now occupies, and would be far more respected by the public at large.

In the following I offer a few contributions of local formulæ, trusting they may be welcome to some readers of the American Journal of Pharmacy:

*Mixtura Rhei et Sodæ.*

R.—Pulv. Rhei,
Sodæ Bicarbonate, . . aa. dr. ij.
Aquæ Menth. pip., . . . . oz. iv.—M.

This, though often prescribed in this city, can only be prepared by the "initiated," because the formula has been kept secret.

*Potio Riveri.*

R.—Potass. Carb. depur., . . . dr. j.
Acid. Citrici, . . . . . grs. lij.
Aquæ, . . . . . . oz. ij.—M.

This preparation corresponds rather closely to our "neutral mixture," and it is often prescribed under the above title by German physicians; but few American pharmacists have been able to prepare it, not knowing the nature of the preparation. If the fraterity would follow this feeble effort in disseminating such knowledge, I am sure it would be productive of very good results, for "In union there is strength."

Hydrate of Chloral.—Joseph R. Beck, M. D., Fort Wayne, Ind. (Phila. Med. Times), is decidedly inclined to the opinion that a dose of fifteen grains is sufficient to commence with in any case. In regard to the frequency of the dose, experience has taught him that it may be safely repeated every two hours for a limited time—say twelve or twenty-four hours, if its effect be not induced sooner.

Absence of Vagina and Uterus.—Dr. Warner reports the following case to the Gynæcological Society of Boston (Gynæcological Journal):

"Mrs. ——, aged twenty, native of Germany, consulted me for sterility. She had never seen any sign of her courses, though she had been married two years. Upon examination, the external genitals were found to be normal, but upon attempting to proceed further, a mere fossa, less than an inch in depth, was found to occupy the location of the ostium vaginæ. A catheter introduced into the bladder came directly into contact with the extremity of the finger within the rectum. Desiring to place the diagnosis beyond a doubt, according to my custom in obscure cases of pelvic tumors, ether was administered upon a later occasion, in company with Dr. Bixby, the sphincter ani ruptured, and the diagnosis fully confirmed by the more extended upward exploration of the pelvis thus permitted."

Pills of Creosote.—The following formulæ are published in Journ. de Pharm. et de Chim., 1871, Oct., p. 276:

| | | |
|---|---|---|
| R.—Creosote, . . . . | | gtt. j. |
| Powd. Soap, . . . . | 0.25, | (gr. iv). |
| Bread crumb, . . | 0.20, | (gr. iij). |
| Lycopodium, . . . | 0.05, | (gr. $\frac{3}{4}$). |
| R.—Creosote, . . . . . | | gtt. iij. |
| Bread crumb, . . . | 0.60, | (gr. ix). |
| Lycopodium, . . | 0.06, | (gr. j(. |
| Mucil. tragacanth, . . . | | q. s. |

Each formula is for six pills, which contain respectively one-sixth and one-half a drop of creosote.

Pills of Carbolic Acid.—

| | | |
|---|---|---|
| R.—Carbolic Acid, . . . . | | gtt. iij. |
| Powd. Soap, . . . | 0.60 | (gr. ix). |
| Lycopodium, . . . | 0.06 | (gr. j). |
| Powd. Tragacanth, . . . | | q. s. |

To make six pills.

The two first ingredients form a semifluid mass, which the lycopodium does not absorb, but which thickens with the tragacanth. —*Ibid.*

A New Excipient for Pills.—J. B. Barnes, writes (Phar. Jour., London,) the following:

"Soluble cream of tartar is a solution of bitartrate of potash in biborate of soda, boracic acid, or biborate of soda and tartaric acid; either of these compounds, when evaporated to the consistence of mucilage, is heavy and adhesive.

Sulphur is generally taken in combination with bitartrate of potash; and the soluble modification of this salt possessing the above-mentioned properties, it suggested to my mind the employment of so appropriate an excipient for the conversion of this substance into pills; and I venture to suggest that pills so prepared might be employed when this substance is required to be taken in doses of between four and twenty grains.

The sulphur pills, prepared respectively with the sublimed and precipitated varieties, contain in each four or five grains, together with one grain in twelve pills of gum tragacanth, and a sufficient quantity of soluble cream of tartar. The pills containing four grains of precipitated sulphur are smaller than it is possible to prepare them with any of the ordinary excipients, being not quite so large as a five-grain compound rhubarb pill, and as hard as a lozenge. When placed in tepid water, the soluble cream of tartar speedily dissolves, and the sulphur is set free.

I propose to call them "sulphur and cream of tartar pills."

I have also prepared five-grain pills of chloral, Dover's powder, nitrate of potash, chlorate of potash, citrate of potash, and gallic acid. The formula used for the chloral pills is as follows:

R.—Hydrate of Chloral, . . . dr. j.
Soluble Cream of Tartar (of the consistence of mucilage), . . gtts. ij.
Gum Tragacanth, . . . grs. ij.

Mix and divide into twelve pills. These require to be kept in contact with lycopodium. They keep their form perfectly and gradually harden; minute glistening particles of the drug have, however, made their appearance on the surface of these pills, and also on the bottle, indicating that they should not be made too long before they are required to be used.

Dimness of Vision Following the Use of Hydrate of Chloral.—E. Burk Haywood, M. D., Raleigh, N. C. (Rich. and Louis. Med. Jour.), writes that he gave hydrate of chloral, in twenty-grain doses, for two weeks, to an old gentlemen complaining of buzzing in the head, pulsation in the epigastrium, and soreness around the waist. At the expiration of two weeks the patient complained of dimness of vision, which, rapidly increasing, he suspected that this remedy caused it, and discontinued its use, when his vision improved, and gradually became as good as it was before using the hydrate of chloral.

FISSURED NIPPLES.—The constant current was used in four cases of excoriated and fissured nipples in nursing women. A small nipple electrode of silver or platina was applied to the abrasions, and into the fissures on or around the nipples for a few minutes. A mild current was used and continued until the diseased part presented a greyish ash color; subsequently it was exposed to the air thirty or forty minutes, to harden or oxidize the affected part. Any moisture that remained was dusted over with dry oxide of zinc. Invariably in the course of twenty-four to forty-eight hours the process of healing was complete.—*Dr. Murry in Med. Record, N. Y.*

SCARLET FEVER.—Dr. E. H. Lewis, (Northwestern Medical and Sur. Jour.) says he found considerable advantage in some cases by using the following prescription:

| | |
|---|---|
| R.—Potass. Chloras, . . . | dr. iss. |
| Tr. Ferri. Chlor., . . | f. dr. ij. |
| Syr. Simple, . . . . | oz. iss. |
| Aq. Fontana, . . . . | oz. iiss.—M. |

S.—Tea-spoonful, every 2 or 3 hours, to a child of 3 to 8 years old.

He gives chlorate potass., in lemonade, during the whole course of the disease, when not using the above.

He has seen great benefit by using a weak solution of chloride of sodium thrown up the nostrils by the posterior nasal syringe.

PERCHORIDE OF IRON IN POST-PARTEM HEMORRHAGE.—Dr. J. T. Johnson, (National Med. Journal) says:

"In the Dublin practice of midwifery, the bold advice of Dr. Robert Barnes, of London, is followed in cases of *post-partem* hemorrhage, in the injection into the uterus of a solution of the perchloride of iron—strength, one part to four parts of water. They inject very cold water into the uterus, taking the usual precaution not to inject air, and if not successful in controlling the hemorrhage, they then use the perchloride of iron. They are much pleased with its effect, and recommend it very strongly."

OVARIOTOMY.—Dr. J. T. Johnson, (National Med. Journal) in referring to his visit to England, said:

"Mr. Wells invited me to witness him perform ovariotomy at the Samaritan hospital, which is practically under his charge. I never before saw an operation of any kind performed so neatly, so quickly, and yet without neglecting a single detail.

In conversation, after the operation, he remarked that 'this was his thirty-third operation of the kind during the year,' and that 'thirty-two previous to this had all *resulted* favorably, and he saw no reason why this one should not.'

I asked him especially about his after-treatment, and told him of the mortality which had attended this operation in our city.

He smiled, and said he 'had no after-treatment to recommend.' In some cases he had not given any medicine, not even opium, unless especially indicated. He said 'too much care was taken of those patients, that they were sometimes killed with over-kindness. He treated symptoms only, and waited for them to arise before he prescribed. He told me the second volume of his book would soon be out, in which he should fully express his views upon the diagnosis, the time to select for the operation, and the after-treatment."

He was very particular to secure all minute bleeding vessels in the omentum and pedicle. Whenever he found a slight oozing, produced by the breaking up of adhesions or from cut surfaces, he would tie a ligature of cat-gut below the bleeding point, and return it into the cavity of the abdomen. I asked him in my innocence how he removed these ligatures, and he smiled again, in a pitying kind of way, and said, 'I never make the attempt. They do no harm. They are composed of animal matter, and are finally absorbed." He said when the profession would use these ligatures and prevent *all hemorrhage* into the cavity of the peritonium they would have fewer inflammations and fatal cases. He had left fifteen of them in the abdomen of one patient and they had made no trouble whatever."

Vaccine Virus in Glycerine.—Dr. A. P. Merrill, New York, (Medical and Surgical Reporter) says:

"For convenience in vaccinating, I take two pieces of glass, say an inch or more in diameter, and make a slight and rough depression in the centre of each by grinding on a small stone. Into this depression I place a crust, or part of one, add two or three drops of glycerine, and then crush the crust by strong pressure. When used, a drop or two of water may be added to soften the solution. I have not been particular to ascertain how long vaccine virus can be preserved in this way; but certainly for months, if not years.

Chlorate of Potassa in Dysentery.—Dr. A. B. Isham, of Cincinnati Ohio, (The Clinic), says that in cases of chronic purulent dysentery, which resisted the usual mode of treatment, he has resorted to the use of chlorate of potassa with succes.s

CHRONIC ENLARGEMENT OF PROSTRATE GLAND.—In this condition, M. Moritan used iodoform as a suppository, one scruple to one ounce of cocoa butter, with great benefit.

SPINAL IRRITATION.—Dr. J. B. Corry, (Northwestern Medical and Surgical Journal) in the treatment of spinal irritation places more reliance in blistering the spine than in any other individual remedy. He finds the majority of cases to be benefited by the following:

R.—Bromid. Potass., . . . . oz. ss.
Fl. Ext. Valerian, . . . oz. ij.
Spts. Ammonia Aronat., . . . oz. j.
Syrup Simplex, . . . . oz. j.—M.

S.—A tea-spoonful three or four times a day.

SULPHO-CARBOLATE OF SODA IN SCARLATINA.—Dr. J. B. Crawford, of Wilkesboro, (Trans. of Med. Soc. of Pennsylvania,) says: "Scarlatina occurred in almost every section of Luzerne county, varying much in severity in different cases and localities, but seldom evincing a marked malignancy of character. In addition to the better-known remedies, the sulpho-carbolate of soda has been used with very satisfactory results in the treatment. I take this occasion to strongly recommend its use to those practitioners who have not yet tested its efficacy in the treatment of this disease. In about thirty cases of scarlatina, of average severity, I have relied almost exclusively upon this article, and have been highly gratified with the results. To a child of three years of age I would give one and a-half to three grains, every two hours, during the continuance of the more severe symptoms of the disease. It is generally readily taken by the patient and well tolerated by the stomach when thus prepared:

R.—Sodæ sulpho-carbolat., . . dr. ij.
Aquæ puræ, . . . f. dr. iv.
Syr. aurant. cort., . . . f. dr. ij.—M.

Sig.—A tea-spoonful to be given once in two to four hours.—*Medical Cosmos.*

OILING URETHRA TO PROMOTE CATHETERISM.—Dr. James C. L. Carson writes to the editor of the Lancet: "Your number for November 11th, contains a letter from Mr. Barrett, recommeding the injection of oil into the urethra previous to the use of the catheter. A far more convenient method of oiling the passage was suggested about a year since in, I think, your journal—namely, to dip the catheter into a vessel containing oil, and whilst it remained in the oil to place the point of the finger of the right hand on the upper end of the catheter, and keep it there till the lower end of the instrument is inserted into the urethra, when the finger may be lifted off the top. On this plan the catheter will lift and carry a sufficient quantity of oil to the urethra; and when the finger is lifted, the oil will flow from the instrument into the canal, and lubricate the stricture. I have tried this plan in several cases of an aggravated character with marked success. Two of these patients were sent to the infirmary for the purpose of being punctured, as the surgeons in attendance, who were well up to their duty, had failed in passing the catheter. I tried the ordinary plan, and failed also; but on using the oil as already described, I succeeded without much trouble."

Suppurative Hepatitis.—Dr. J. L. Hill, (Oregon Med. and Sur. Reporter,) after exhausting every other means in a case of Suppurative Hepatitis, employed the following with immediate benefit, followed by a permanent cure:

| | |
|---|---|
| R.—Ammonia Muriatis, . . . | dr. iij. |
| Tr. Cimicifuga, - . . | oz. iss. |
| Aquæ, . . . . . | oz. ij.—M. |

S.—A tea-spoonful four times a day.

Acid Dyspepsia.—That form associated with flatulence is a very common one of dyspepsia. This form of dyspepsia with acidity cannot be counter-acted by alkalies, but by phosphoric, hydrochloric, or lactic acids,—the disease being due to want, or dilution of, the gastric juice.

Dyspepsia—Associated with sluggishness of the large intestines and obstinate constipation, is marvellously controlled by belladonna. Small doses should be given at first, morning and evening, to be gradually increased. Should the belladonna fail alone, a tea-spoonful of castor oil may be given, simultaneously, with it.

Calabar Bean in Excitement of Cerebro-Spinal Nervous System.—Prof. N. S. Davis (Chicago Medical Journal) says: I am satisfied that in some forms of irritation, or morbid excitement of the cerebro-spinal nervous system, especially accompanied by muscular rigidity, we may find the Calabar Bean a valuable remedy."

Has it ever been employed in Cerebro-Spinal Meningitis?—Eds.

PERIMETRITIS.—Prof. Byford reports a case of Perimetritis (Chicago Medical Examiner), and in relating its history, he says:

"She married thirteen years ago, at the age of sixteen. Her health was perfect until the birth of her first child, but ever since she has suffered from dysmenorrhœa and leucorrhœa between the molimena. She had fever, pain in the back, hips and abdomen, pain in urinating, nausea and vomiting. The fever was considered to be an intermittent by a physician who saw her before she came to the hospital. The house physician here prescribed paregoric and nitre, on account of the pain in the bladder, believing she had cystitis, and for the nausea and vomiting he gave her carbolic acid dissolved in glycerine and water. These medicines effectually relived the symptoms before the patient was seen by the Professor. On examining her, he at once recognized a case of perimetritis. A lump, hard as bone, and very tender under pressure, was felt in the left iliac region, extending diagonally into the other iliac region. By a vaginal examination, a hard tumor was found filling the pelvis and pressing against the bladder. The consequent inflammation explains the pain in urinating; but, besides this tumefaction of the uterus, the pelvic cavity was also filled by a fibrinous exudation.

A mistake in diagnosis is liable to be made by almost any one, especially since examination is so frequently neglected. The chills, fever and perspiration occurring every morning, made up apparently a good case of intermittent fever for the physician who first saw her; but the dysury and pains ought to have induced an examination. External exploration should be made in search of hard, resisting masses. Hard tumors can be distinguished by their giving dullness on percussion, whereas the intestines gives resonance on percussion. Of course, a vaginal examination should be made. The extreme frequency of these cases is often overlooked, because examination is neglected.

Fomentations of aconite and arnica leaves were directed, and likewise poultices of corn meal or flax-seed. The mixture of muriate of ammonia, tartarized antimony and morphia, was also ordered to be given four times a day. She is improving rapidly, and is about ready to go home. At present, two and a-half weeks after her entrance into the hospital, examination shows that the pelvic mass is gone, and there is no tumefaction, except in the uterus, which is moveable."

A CASE OF GLANDERS in a man, cured by the internal use of carbolic acid, is reported in the proceedings of the Minnesota State Medical Society.

Charcoal in Ulcer of Stomach.—Dr. J. Farrar (London Lancet), after employing every remedy he could devise, in a case of ulcer of the stomach, succeeded in curing his patient by the internal use of animal charcoal. He gave half-teaspoonful doses three times a day, to be taken, if possible, mixed with a little cold water—just enough to form it into a bolus—and taken half an hour or more before eating.

Aconite Productive of Uterine Irritation.—Dr. Patterson (Medical & Surgical Reporter—proceedings of Tompkins county Med. Soc., N. Y.,) remarked that he believed aconite (tincture), given in more than one-drop doses, would produce serious uterine irritation, if continued any length of time, in many cases. In this way he accounted for many cases of puerpural eclampsia in the hands of homeopathics.

Monobromized Camphor.—Prof. Denefee, of Ghent, has for over two years employed a combination of camphor and bromine, —in the form of a crystalized substance which is called as above —which he esteems entitled to attention as an excellent sedative to the nervous system. He prescribes it in delirium tremens, in the form of pills—seventy grains being made into thirty pills—of which one may be given every hour, until twenty are taken. For three days longer from forty-five to sixty grains may be given, the quantity being diminished from forty-five to thirty grains daily for a week longer.

Gelseminum in the Treatment of Irritable Bladder.—Dr. W. Scott Hill, Augusta, Maine, (Am. Jour. Med. Sciences, Jan., 1872), gives five cases of "irritable bladder" successfully treated with gelseminum. In all the cases the same symptoms were present, namely: frequent calls to void the urine, which was small in quantity, often passed *guttatim*, and excessive pain attending micturition. He used Tilden's Fluid Extract of Gelseminum. His usual prescription was:

| | |
|---|---|
| R.—Potassii Bromid., . . . | gr. iv. |
| Potass. Carbon., . . . . | gr. iij. |
| Fluid Ext. Gelsem. . . . | m. x. |
| Aquæ., . . . . . . | oz. ij.—M. |

S.—This quantity every 4 or 6 hours.

Cotton Root in Menstrual Suppression and Dysmenorrhœa. Dr. Robt. E Peyton, Fauquier, Va., (Richmond & Louisville Med. Jour.) speaks highly of cotton root in these disorders. In a severe case of Dysmenorrhœa, he gave a tablespoonful of the fluid extract, with a little water three times in twenty-four hours—commencing one week before the menstrual period; and after the occurrance of pain four or five times a day, until they subsided.

PODOPHYLLIN.—In an article on the uses of this remedy, in the Practitioner, Dr. Charles Phillips says: "The vomiting and diarrhea which occur in gastro-enteric inflammation are often arrested, and with rapidity, by the exhibition of a few doses of the one-sixth to the one-twelfth of a grain every four or six hours. In the remittent fevers of children, where there is much heat of skin, headache with delirium, a quick and full pulse; dry, brown, and furred tonge; nausea, or vomiting of bilious matter; pain or uneasiness in the stomach; sleeplessness, with a general sense of weariness, and a grinding of the teeth during sleep; podophyllin, taken in doses of one-tenth of a grain every four hours, will often cut short those various symptoms in a very remarkable manner. The effect is much improved by the exhibition of occasional doses of aconite. When the stools of youug children and infants have been white or clay-colored, podophyllin has frequently brought the bile into their motions by administrations in doses of one-tenth to one-twentieth of a grain every six hours, persevering in the use of it for a short period. In these cases it also regulates the bowels. Prolapsus of the rectum in children may also sometimes be removed by the administration of similar doses of podophyllin every night and morning. In dyspepsia and in hepatic derangements, characterized by loss of appetite, acid regurgitation, putrid taste in the mouth, flatulence, and a tendency to constipation or to diarrhœa, one-tenth of a grain of podophyllin, exhibited night and morning, will frequently induce the best results.—*American Practitioner.*

COTTON ROOT (GOSSYPIUM) AS AN ANODYNE.—Dr. Robt. E. Peyton, (Richmond & Louisville Med. Jour.), reports a case far gone with consumption, troubled by distressing nervous symptoms, attended with suffocative cough, and apprehension of immediate dissolution. McMunn's Elixer of Opium proving unsatisfactory, he was given a teaspoonful of the fluid extract of cotton root with much relief, and a few repetitions gave him a comfortable night.

SEMINAL EMISSIONS.—In his Clinic, Prof. Gross (Med. & Surg. Reporter), in speaking of seminul emissions, says:

It is well for a patient who suffers from an affection of this kind, to make a light supper; and he should see that the bladder was well emtied before retiring, as well as promptly voiding the urine that collects during the night, so soon as he is made aware of its presence. All errors of secretion and digestion should be corrected; and if morbid sensibility of the urethra exists, it should be injected with a solution consisting of three grains of the acetate of lead to an ounce of water. If the undue sensibility exists at the outlets of the seminal ducts in the prostatic urethra, cauterize them, using for the purpose the operator's urethral paste-caustique. A sound was introduced into the patient's urethra, but no undue sensibility was excited. He has some dyspepsia. His emissions generally occur in the morning, about "the time of the crowing of the cock." This patient was ordered to dash cold water against his genial apparatus before getting into bed; to sleep upon a mattrass, and to take internally

R.—Elix. cinchonæ calis. . . dr. jss.
Acid nitricum dil., . . . gtt. viij.
Strycniæ sulph., . . . gr. 1-16.—M.

S.—Take the above quantity three times daily.

Also:

R.—Morphiæ sulphatis, . . gr. 1-4.
Cocoa butter, . . . . q. s.—M.

Ft. in suppository, No. 1.

S.—Introduce into the bowel at bed-time.

Also:

R.—Liq. plumbi subacetatis, . . dr. j.
Aquæ, . , . . . oz. x.—M.

S.—Inject into the urethra twice in the 24 hours.

Macrotys Racemosa.—Dr. J. M. Scudder, (Eclectic Med. Jour.) writes of this drug as follows:

For years I have employed Macrotys as a specific in rheumatism, and with excellent success. Not that it cures every case, for it does not, neither would we expect this, for this would be prescribing a remedy for a name. Rheumatism may consist of varied pathological conditions, though in all there is the special lesion of the nervous system, which characterizes the disease. In one case we find the indications for the use of an Acid prominent, and this becomes a remedy for rheumatism. In another there are symptoms showing the need of Alkalies, and they prove curative.

Macrotys influences the nervous system directly, relieves rheumatic pain, when not the result of inflammation, and probably corrects the diseased condition (formation of lactic acid ?) which gives origin to the local inflammatory processes. Thus in the milder cases, where the disease has not localized *itself* as an inflammation, Macrotys is very speedy and certain in action. In rheumatic fever it is also positive in its action, and with the special sedatives gives excellent results. Where rheumatism has localized itself in inflammatory process, all the benefit we attain from it is, that we remove the cause, and hence the reason for a long continuance of the inflammation.

It is a remedy for all pains having a rheumatic character, and for this we prescribe it with the best results. Those cases which go under the name of reumatic-neuralgia, are very speedily relieved by it. In somes cases the pains of weeks' duration disappear in a single day. Whilst the continuance of the remedy will not unfrequently effect a cure in these cases, in many it will require the additional means necessary to give healthy functional activity to some organ or part especially impaired.

The Macrotys influences directly the reproductive organs. This influence seems to be wholly upon the nervous system, relieving irritation, irregular innervation, and strengthening *normal* functional activity. For this purpose it is unsupassed by any agent of our Materia Medica, and is very largely used.

Like all other *direct* remedies, it may be employed in any case, no matter what name the disease may have in our nosological classification, if the condition of the nervous system calls for it. The heavy, tensive, aching pains are sufficiently characteristic and need not be mistaken. So prominent is this indication for the remedy, in some cases (not rheumatic), that I give it with a certainty that the entire series of morbid processes will disappear under its use.

Diagnosis of Bright's Disease.—Dr. Charlton (British Med. Journal), has found creasote so uniformly successful in checking the vomiting in Bright's disease, that he has diagnosticated this malady, where other symptoms were absent, by the cessation of vomiting under this remedy. As another diagnostic sign, he states that tenderness on pressure of the pneumogastric nerve, in its course through the neck, is evidence of inflammatory disease of some of the organs to which it is distributed. If only one side is affected, the nerve on that side will alone be tender.—*Canada Medical Journal.*

Vapor of Ammonia in the Treatment of Hooping-cough.—Mr. John Chatham states (Brit. Med. Journal, Sept. 16, 1871) that in cases of hooping-cough in the last stage (that is, after the third week) which he has had recently, he has had one ounce of the strongest liquid ammonia put into a gallon of boiling water in an open pan, and the steam kept up by means of half a brick made red-hot throughout and put into the boiling water containing the ammonia, the pan placed in the centre of a room, into which the patients were brought as the ammoniated steam was passing off. "This method was used in the evening, just before bed-time; and it has been so efficacious," he says, "in abating the spasmodic attack, and after three or four days terminating the malady, that I cannot overestimate the great value of this mode of inhaling the ammonia as a therapeutic agent in tranquilizing the nervous system in hooping-cough."

Treatment of Chlorosis.—M. Delioux de Savignac proposes, in the *Bulletin de Therapeutique*, the following formula as being likely to fulfil most completely the ordinary indications of treatment in chlorosis: tartrate of iron and potassa, ten grammes; powdered castor, two grammes; powdered saffron, one gramme; to be made into a mass with Venice turpentine and divided into a hundred pills. At first, three pills are to be given daily, and the number is to be gradually increased to six or nine; care being taken to maintain a free action of the bowels without producing diarrhœa. The pills are to be taken three times daily; early in the morning and at luncheon and dinner. M. Delioux de Savignac explains that the aloes is intended to obviate the constipation frequently met with in chlorotic patients: the castor and saffron to relieve flatulent distension of the abdomen; while the turpentine exercises a beneficial influence on the leuchorrœa. If the aloes act too energetically, it may be replaced by rhubarb; or, if constipation remain obstinate, a little jalap, scammony, or gamboge may be added. If the bowels be already free, M. Delioux omits the purgative altogether. When the turpentine produces gastric disorder, or colic with or without diarrhœa, balsam of Peru may be substituted for it. The use of the pills is contraindicted in chlorosis attended with menorrhagia.—*Brit. Med. Journal*, Sept. 30, 1871.

Anhydrous Glycerine.—M. Eberhard has called attention to the power possessed by absolutely anhydrous glycerine of withdrawing water by an exosmotic process from tissues to which it is applied. Marion Sims sometime ago demonstrated that a ball of lint dipped in glycerine and applied to a freely suppurating surface arrests the secretion. Turst has also applied the glycerine plug in a large number of cases of fluor albus, and M. Eberhard states that he has been very successful in applying the same means in similar cases.—*The Druggists' Circular*, Oct. 1871.

