# Correction

**Published:** 1872-04

**Authors:** 


### PART III.

### Editorials, Correspondence and Miscellaneous.

### CORRECTION.

Dr. Hodges, of Arkansas, writes to inform us that a mistake occurred in the February edition, in regard to the number of pills to be made from the formula of his "Sovereign Anti-Bilious Pill." The mass should be divided into sixty (60) pills. We trust our friends will make the correction in their copies. We will be pleased to hear from Dr. H. again.

